# Laminin promotes differentiation of rat embryonic stem cells into cardiomyocytes by activating the integrin/FAK/PI3K p85 pathway

**DOI:** 10.1111/jcmm.14264

**Published:** 2019-03-25

**Authors:** Duo Wang, Yumei Wang, Huan Liu, Chang Tong, Qilong Ying, Agapios Sachinidis, Li Li, Luying Peng

**Affiliations:** ^1^ Key Laboratory of Arrhythmias, Ministry of Education, Shanghai East Hospital Tongji University School of Medicine Shanghai China; ^2^ Research Center for Translational Medicine, Shanghai East Hospital Tongji University School of Medicine Shanghai China; ^3^ Department of Pathology and Pathophysiology Tongji University School of Medicine Shanghai China; ^4^ Eli and Edythe Broad Center for Regenerative Medicine and Stem Cell Research at USC, Department of Stem Cell Biology and Regenerative Medicine, Keck School of Medicine University of Southern California Los Angeles California; ^5^ Institute of Neurophysiology and Center for Molecular Medicine University of Cologne Cologne Germany

**Keywords:** cardiomyocyte differentiation, FAK, integrin, laminin, PI3K, rat embryonic stem cell

## Abstract

The generation of germline competent rat embryonic stem cells (rESCs) allows the study of their lineage commitment. Here, we developed a highly efficient system for rESC‐derived cardiomyocytes, and even the formation of three‐dimensional (3D)‐like cell clusters with cTNT and α‐Actinin. We have validated that laminin can interact with membrane integrin to promote the phosphorylation of both phosphatidylinositol 3‐kinase (PI3K) p85 and the focal adhesion kinase (FAK). In parallel, GATA4 was up‐regulated. Upon inhibiting the integrin, laminin loses the effect on cardiomyocyte differentiation, accompanied with a down‐regulation of phosphorylation level of PI3K p85 and FAK. Meanwhile, the expression of Gata4 was inhibited as well. Taken together, laminin is a crucial component in the differentiation of rESCs into cardiomyocytes through increasing their proliferation via interacting with integrin pathway. These results provide new insights into the pathways mediated by extracellular laminin involved in the fate of rESC‐derived cardiomyocytes.

## 1 INTRODUCTION

The rat (*Rattus norvegicus*) laboratory model is one of the most widely used animal models in scientific community.^1^ As an experimental model in physiological and pharmacological studies, the rat model has striking advantages compared with mouse. The rat model is well characterized and has been applied in many fields, including cardiovascular system, pulmonary, pharmacology, etc.^2^, ^3^ However, rat models based on genetic manipulation have been lacking until authentic germline competent rat embryonic stem cells (rESCs) were generated.^4^, ^5^ Both transgenic and gene‐engineered rat models have been showed with obvious advantages over the mouse ones. For example, the p53‐knockout rats are susceptible to cancer without additional tumour induction and more likely to suffer hepatic hemangiosarcoma with higher morbidity of developing lymphoma than the p53^‐/‐^ mouse does.^6^, ^7^ In addition, the deletion of LDL receptor (Ldlr) in rat was found to have a profound increase in plasma levels of total cholesterol like Ldlr^‐/‐^ mice. The plasma triglyceride levels in Ldlr^‐/‐^ rats were 1.8‐fold higher than of the wild‐type, whereas no changes in Ldlr^‐/‐^ mice has been observed.^8^ Therefore, the similarity of some hallmark physiological functions between rat and human makes the rat model more suitable than the mouse model for heart disorders are more relevant to human conditions.

Adult human cardiomyocytes normally do not proliferate, which is a major obstacle to understand the underlying pathogenesis of heart diseases and to identify cardiotoxic effects in drug screening. Based on physiological similarities between the rat and human, it is potential to develop in vitro differentiation approach to generate functional cardiomyocytes from rESCs.^9^ Using standard differentiation medium (DM), an rat embryoid body (rEB)‐based cardiomyocyte differentiation method has been developed by gradually reducing the concentration of 2i at early differentiation stage.^9^ While this in vitro system is rudimentary, only 6.6 ± 2.0% cTNT‐positive cells were obtained. Therefore, there is still a need to establish a more robust rESC‐derived cardiomyocyte methodology.

Extrinsic cellular factors are involved in stem cell lineage specification, and increasing evidence indicates that the nanoscale geometry/topography of the extracellular matrix (ECM) impact stem cell fate. The ECM can set up extracellular environment, thereby, inducing the cell lineage by providing ligand interactions with stem cell surface receptors.^10^ Moreover, the ECM can also create a 3D environment for stem cell attachment, growth and differentiation. With the development of tissue engineering technologies, special ECMs have been purified and widely used for in vitro cell differentiation^11^ as well for therapies in vivo.^12^ Recently it was demonstrated that the ECM protein named Agrin binds to dystrophin glycoprotein complex, causing YAP release and translocation into the nucleus thereby promoting myocardial cell proliferation.^13^ Some components of ECMs, including gelatin, laminin and matrigel have been widely used to create in vitro cardiomyocyte differentiation from both mESC and hESC. This mixture generates a different ground state for rESCs, where the role of ECMs on rESCs attachment and differentiation remains elusive. Integrins as cell adhesion receptors can mediate the attachment of cells to the extracellular matrix thereby promoting cell‐cell interactions.^14^ In vertebrates, integrins are composed of 18 α‐subunits and 8 β‐subunits, which can form 24 different α/β heterodimeric members.^15^ The extracellular domain of integrin can interact with ECMs such as laminin, fibronectin, collagen and vitronectin while the intracellular region can control the corresponding pathways by regulating intrinsic signalling factors.^16^, ^17^ However, how integrins mediate the differentiation process from rESCs still needs to be clarified.

In our present work, we evaluated the effects of different ECMs on cardiomyocyte differentiation from rESCs. Overall, laminin was found to promote rEB differentiation and interact with integrin to affect PI3K/FAK phosphokinase activity. Through screening differentiation conditions, a highly efficient system was developed for establishing rESC differentiation into cardiomyocytes.

## 2 MATERIALS AND METHODS

### 2.1 rESC derivation and propagation

Rat embryonic stem cells were derived and propagated according to a modified methodology as described previously.^18^ Briefly, Dark‐Agouti (DA)‐Sprague Dawley (SD) rat blastocysts were flushed out with N2B27 medium from the uteruses of E4.5 pregnant rats. The single blastocyst was transferred into each well of 24‐well plates that were seeded with the mouse embryonic fibroblasts (MEF) feeder layers as reported previously.^19^ The culture medium included 2i medium: N2B27 medium supplied with 3 μmol/L CHIR99021 (Selleckchem, Houston, TX) and 1 μmol/L PD0325901 (Selleckchem, Houston, TX). After 4‐5 days, the outgrowths of blastocysts were digested by 0.025% trypsin‐EDTA solution (Gibco, Carlsbad, CA) and seeded in the MEF‐2i conditions as DASDC1 line or DASDC2 line. DAC8 line of rESCs was used as control experiment. Detailed methods are described in Supplemental Experimental Procedures. The usage and treatment of rats were approved by Animal Experimental Ethics Committee of Tongji University School of Medicine (TJLAC‐015‐035).

### 2.2 rEB formation and differentiation

Rat embryonic stem cells were trypsinized into single cells in the cellular matrix (CM) with 0.75 μmol/L CHIR9902, 0.1 μmol/L PD0325901 and 10 μmol/L Y27632 (Selleckchem, Houston, TX), then diluted into 2.0 × 10^5^ cells/mL. The cell drops (20 μL each drop) were hung for 2 days for forming the rEBs, which were then harvested and transferred into 50× laminin (Gibco, Carlsbad, CA)‐coated 6‐well plates, and cultured in the CM with 0.75 μmol/L CHIR9902 and 0.1 μmol/L PD0325901. One day later, the attached rEBs were grown in fresh DM or DIF medium. The details of regents and methods are described in Supplemental Experimental Procedures.

### 2.3 Quantitative real‐time PCR

Quantitative real‐time PCR (qRT‐PCR) was performed as described previously.^20^ The details of method are described in Supplemental Experimental Procedures and the related primers are listed in Table S1.

### 2.4 Western blot

Total protein was extracted with ice‐cold RIPA buffer with protease inhibitor (Biotool, Houston, TX) and protein phosphatase inhibitor (Biotool). Proteins were separated with 10% SDS/PAGE gel. Whole information about procedures and antibodies are listed in Supplemental Experimental Procedures and Table S2 respectively.

### 2.5 Immunostaining

The major experimental steps are described in accordance with the previous description.^21^ The rest of the information related to the assay is described in Supplemental Experimental Procedures and in the Table S2.

### 2.6 Proliferation activity assay

Cell counting kit‐8 (Beyotime, Shanghai, China) was used to detect the proliferation of differentiating cells from rESCs. The detailed information is provided in Supplemental Experimental Procedures.

### 2.7 Beating cell culture

Cell drops (2.0 × 10^5^ cells/mL) were placed onto lids of culture dishes for 2 days to form rEBs (20 μL each drop). The rEBs were then transferred into the 24‐well plates treated with 50× laminin for 24 hours before application. At day 3 of differentiation, the cells were cultured in DIF medium and DM medium respectively.

### 2.8 Statistical analysis

Student's *t *test (two‐tailed) was used to evaluate the statistical significance, and the error bar represents the standard error of mean (SEM) of three independent experiments. Statistical differences were considered significant with a value of *P* < 0.05.

## 3 RESULTS

### 3.1 Specific culture conditions for efficient formation of rEBs

**Figure 1:**
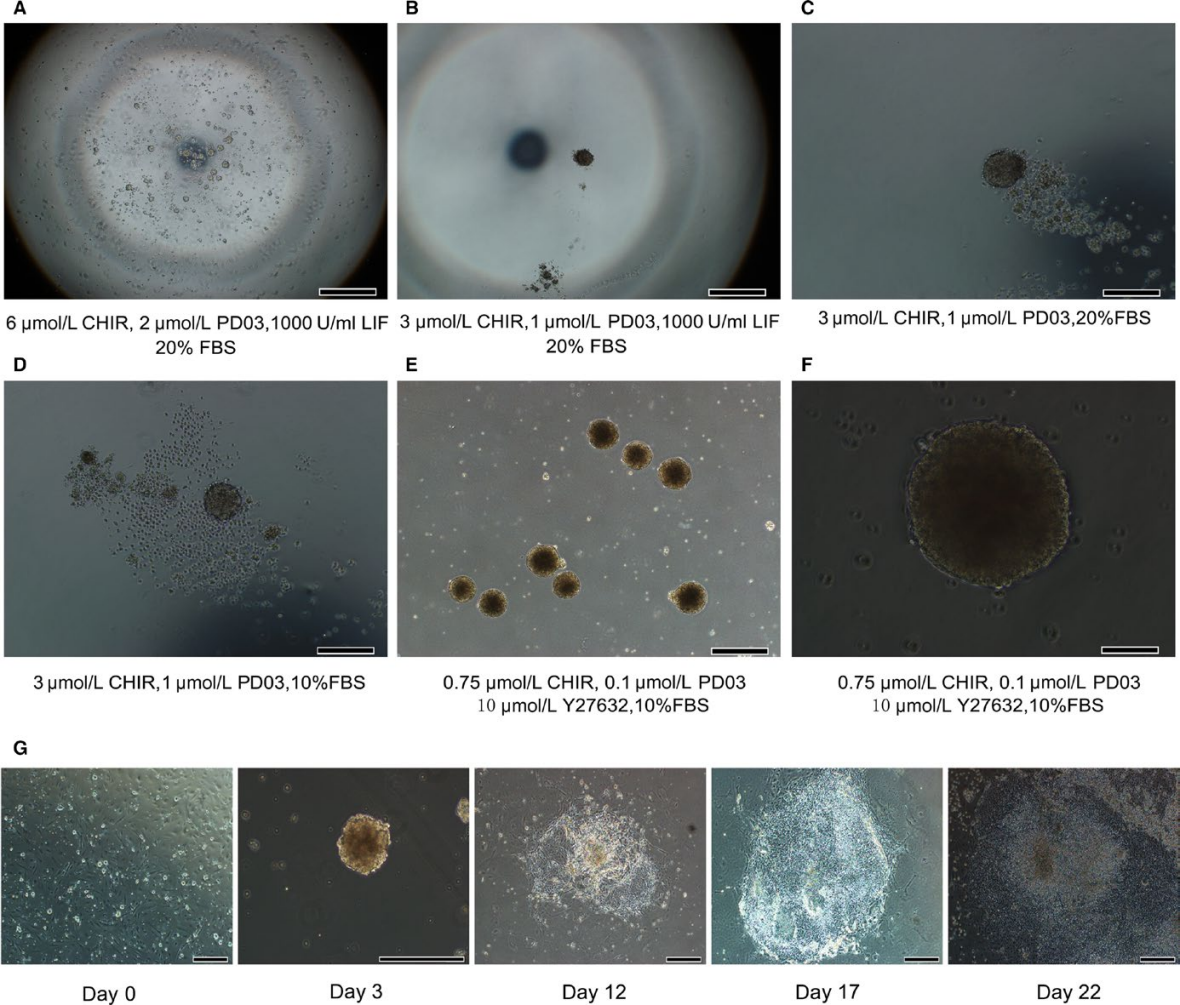
Specific culture conditions for efficient formation of rEBs. A‐E, The rEB morphology after 2 days hanging droplets under different culture condition (scale bar: 500 μm). F, Zoom in figure (E) (scale bar: 200 μm). G, The different cell morphology during rESC differentiation (scale bar: 500 μm)

In order to address the need for the optimal conditions for rEBs formation at an early stage of differentiation, different culture medium conditions were tested. In general, the rESCs need to be cultured in the medium containing the GSK3 inhibitor, CHIR99021 (CHIR) and the MEK inhibitor, PD0325901 (PD03). Combination of the two‐inhibitors (2i) effectively maintains pluripotency but there were some differences in culture conditions between rESCs and mESCs. Mouse embryonic fibroblast (MEF) feeder cells required for co‐culturing with mESCs can be replaced by the cytokine leukaemia inhibitory factor (LIF) while rESCs must be cultured with MEF feeder cells.^5^, ^22^ LIF promotes the self‐renewal of mESCs through stimulation of Janus‐associated kinase (JAK) and activation of signal transducers and activators of transcription (STAT3).^23^ These observations would suggest differences in the signal pathway of differentiation initiation between rESC and mESC. To identify the optimal conditions of rEBs formation, the E4.5 rat blastocysts were isolated and then the rESC cell line was established as described previously^18^ by detecting the pluripotent marker OCT4 (Figure S1). For the formation of rEBs, the hanging droplet strategy was applied. The medium containing 6 μmol/L CHIR, 2 μmol/L PD03 and 1000 U/mL LIF failed to induce the aggregation of rEB from rESCs (Figure 1A). Rat EBs were then generated by reducing the concentration of CHIR and PD03 in the medium but the shape of the rEBs was observed to be irregular (Figure 1B). Moreover, the medium without LIF induced the formation of rEB along with a large number of dead cells (Figure 1C). In addition, 20% of foetal bovine serum (FBS) plays a significant role in cardiomyocyte differentiation from mESCs and hESCs,^24^, ^25^ but really impaired the normal state of rEBs in 2i medium during the course of hanging droplets (Figure 1D). To optimize the differentiation conditions, 10% FBS with low concentration of 2i (0.75 μmol/L CHIR and 0.1 μmol/L PD03) and 10 μmol/L Y27632 in medium was used to induce rEBs formation and inhibit cell death (Figure 1E,F). However, using the modified protocol^9^ to generate rEB still resulted in slow cell growth, cell death and delayed beating of cardiac‐like cells at day 22 (Figure 1G and Video S1).

### 3.2 Laminin promoted rEBs proliferation and differentiation

**Figure 2:**
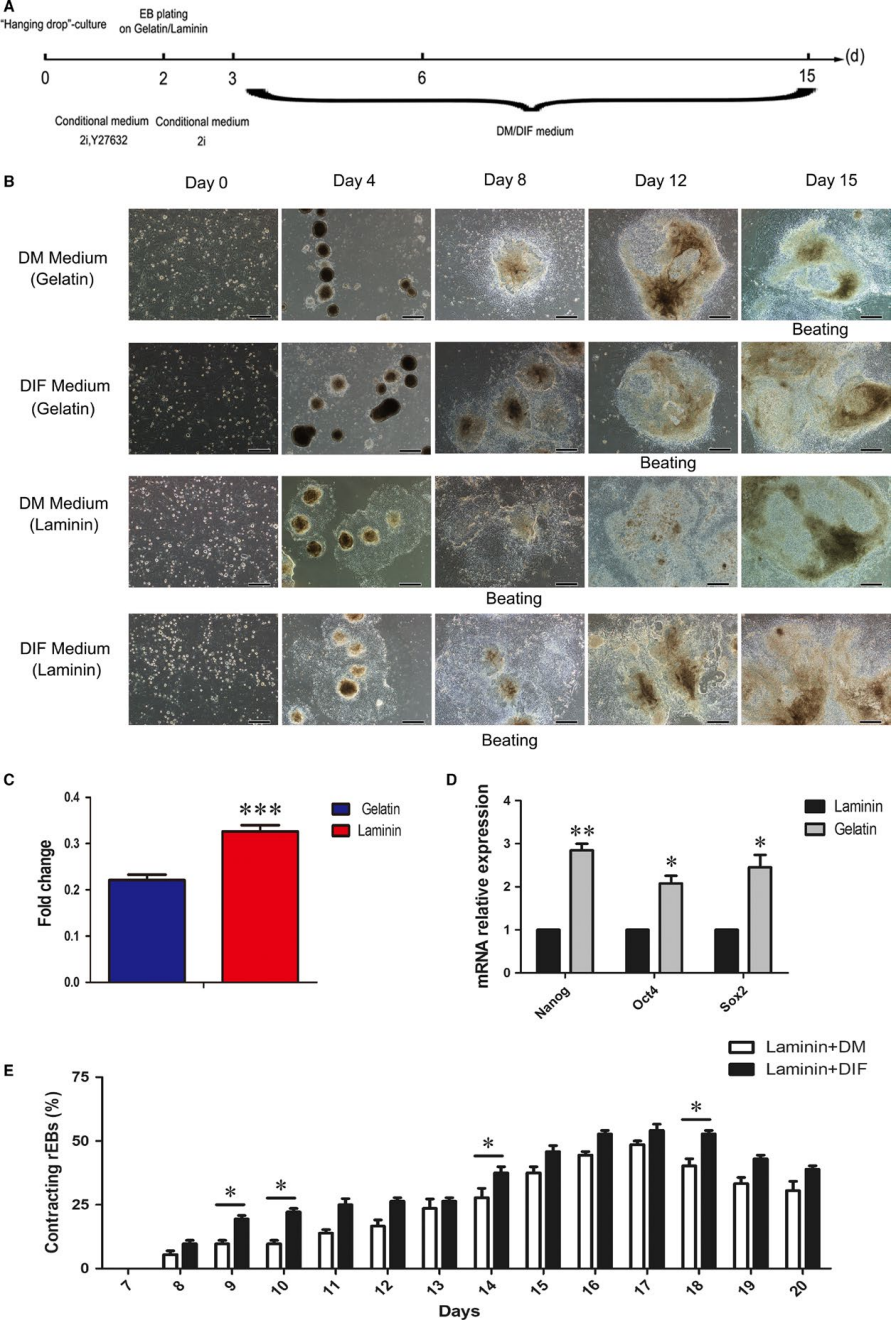
Optimization of extracellular matrix for rEB differentiation and proliferation. A, Schematic diagram of the differentiation procedure. B, The morphology of cells under different culture media and extracellular matrix treatment conditions (scale bar: 500 μm). C, Results for cell proliferation determined by the CCK‐8 assay (n = 6), mean ± SEM, ***P < 0.001. D, Expression of Nanog, Oct4 and Sox2 during differentiation at day 4 (n = 3), mean ± SEM, *P < 0.05, **P < 0.01. E, The beating ratio variation tendency, mean ± SEM, *P < 0.05

To further identify the optimal differentiation conditions for rESCs to cardiomyocytes, we first compared the effects of gelatin and laminin, which have been suggested to facilitate cardiomyocyte differentiation as supportive ECMs.^26^, ^27^ Most of the rEBs generated by the hanging droplet methodology (Figure 2A) and cultured in gelatin coated dishes fail to attach (Figure 2B). We found that laminin‐induced rESC could induce the cardiomyocytes to beat on day 8 of differentiation, and the medium harbouring Laminin/differentiation‐IMDM‐FBS (DIF) produced 55.3% of beating rEBs derived from rESCs (Figure 2E) Interestingly, rEBs cultured in laminin‐coated dishes attached and proliferated well (Figure 2B). Further quantification of the proliferation using the CCK‐8 assay showed that the proliferation rate of rEBs at day 4 was increased as a result of laminin treatment (Figure 2C). These findings indicated a stronger effect of laminin to promote cell proliferation in comparison to gelatin. Moreover, the expression levels of pluripotency genes such as Nanog, Oct4 and Sox2, were significantly reduced in differentiated cells in the presence of exogenous laminin (Figure 2D). These results showed a critical role of laminin on rEB differentiation and proliferation.

### 3.3 Laminin and DIF medium promoted cardiomyocyte differentiation of rESCs

**Figure 3:**
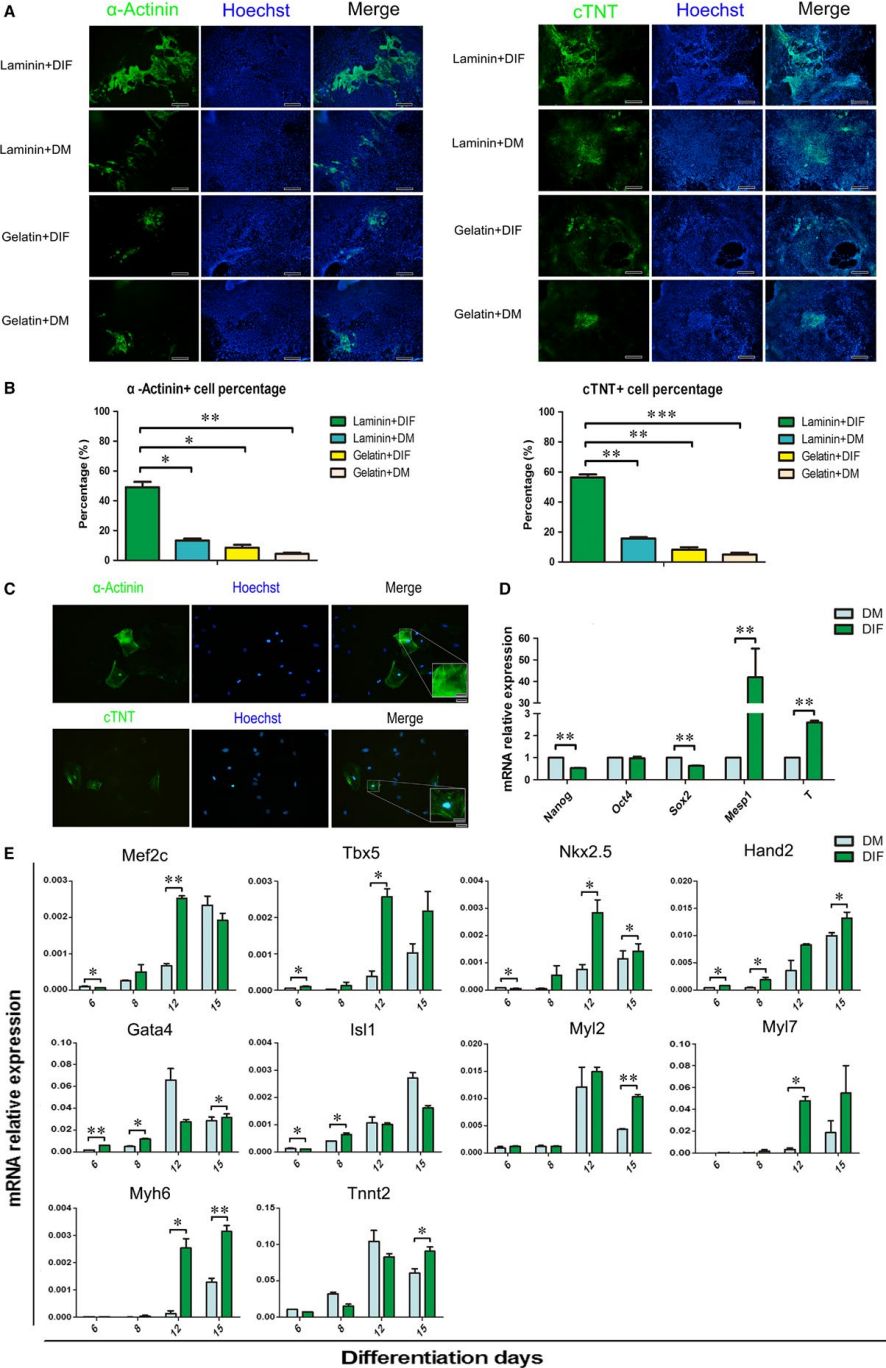
The cardiomyocyte morphology differentiated from rESCs. A, Immunofluorescence results of α‐actinin and cTNT expression in the cardiomyocyte derived from rESCs at day 15 (scale bar: 200 μm). B, The quantification of the immunofluorescence results (n = 3), mean ± SEM, *P < 0.05, **P < 0.01, ***P < 0.001. C, The cardiomyocyte morphology differentiated from rESCs (scale bar: 50 μm; the magnification region scale bar: 16.6 μm). D, The mRNA levels of pluripotent gene markers at differentiation day 6 (n = 3), mean ± SEM, **P < 0.01. E, The mRNA levels of cardiomyocyte‐related genes during cardiomyocyte differentiation under DM/DIF culture medium condition (n = 3), mean ± SEM, *P < 0.05, **P < 0.01

To further check whether laminin can promote cardiomyocyte differentiation and to screen the optimal culture medium for the process, α‐Acinin^+^ and cTNT^+^ cells at day 15 under different conditions were quantified. The percentage of differentiated cardiomyocytes in the presence of laminin was significantly higher than that cultured with gelatin (Figure 3A,B) and the beating rEBs also showed an upward trend compared with that induced by DM (Figure 2E). Although DM of mESC has been used to differentiate cardiomyocyte from rESCs, the cardiomyogenic efficiency was limited.^9^ Given the potential role of serum and ascorbic acid on cardiomyocyte differentiation,^25^ a medium modified by replaced DMEM/F12 with IMDM which harboured 15% of serum and 0.05 mg/mL ascorbic acid was set up as differentiation‐IMDM‐FBS (DIF) medium. Indeed, the DIF medium provides more effective differentiation environment as the proportion of a‐Actinin^+^ and cTNT^+ ^cells in the presence of laminin was increased to 49% and 56.3% respectively (Figure 3A,B). The cardiomyocytes also exhibited a typical sarcomeric structure as shown (Figure 3C). To further evaluate the effect of DIF medium, the expression status of pluripotent‐ and cardiomyocyte‐specific gene markers in the differentiating cells was proved. These results showed that the expression of Nanog and Sox2 was inhibited and as expected Mesp1, T and Gata4 genes were up‐regulated at day 6 of differentiation in DIF (Figure 3D,E). We also found that Mef2c, Tbx5, Nkx2.5, Myl7 and Myh6 were markedly up‐regulated in the cells differentiated with DIF medium and up‐regulation occurred earlier in comparison to DM condition (Figure 3E). In addition, the cardiomyocyte markers, including Nkx2.5, Hand2, Gata4, Myl2, Myh6 and Tnnt2 were up‐regulated at day 15 under the DIF‐culturing conditions (Figure 3E). Under laminin/DIF culturing condition, the beating was firstly observed at day 8 and the areas of beating cells were also significantly improved in compared with previous report (Video S1 and Video S2). These results suggested that the laminin/DIF culturing conditions are optimal for an efficient differentiation of rESCs to cardiomyocytes.

### 3.4 Laminin mediates integrin/PI3K/FAK axis to promote cardiomyocyte differentiation

**Figure 4:**
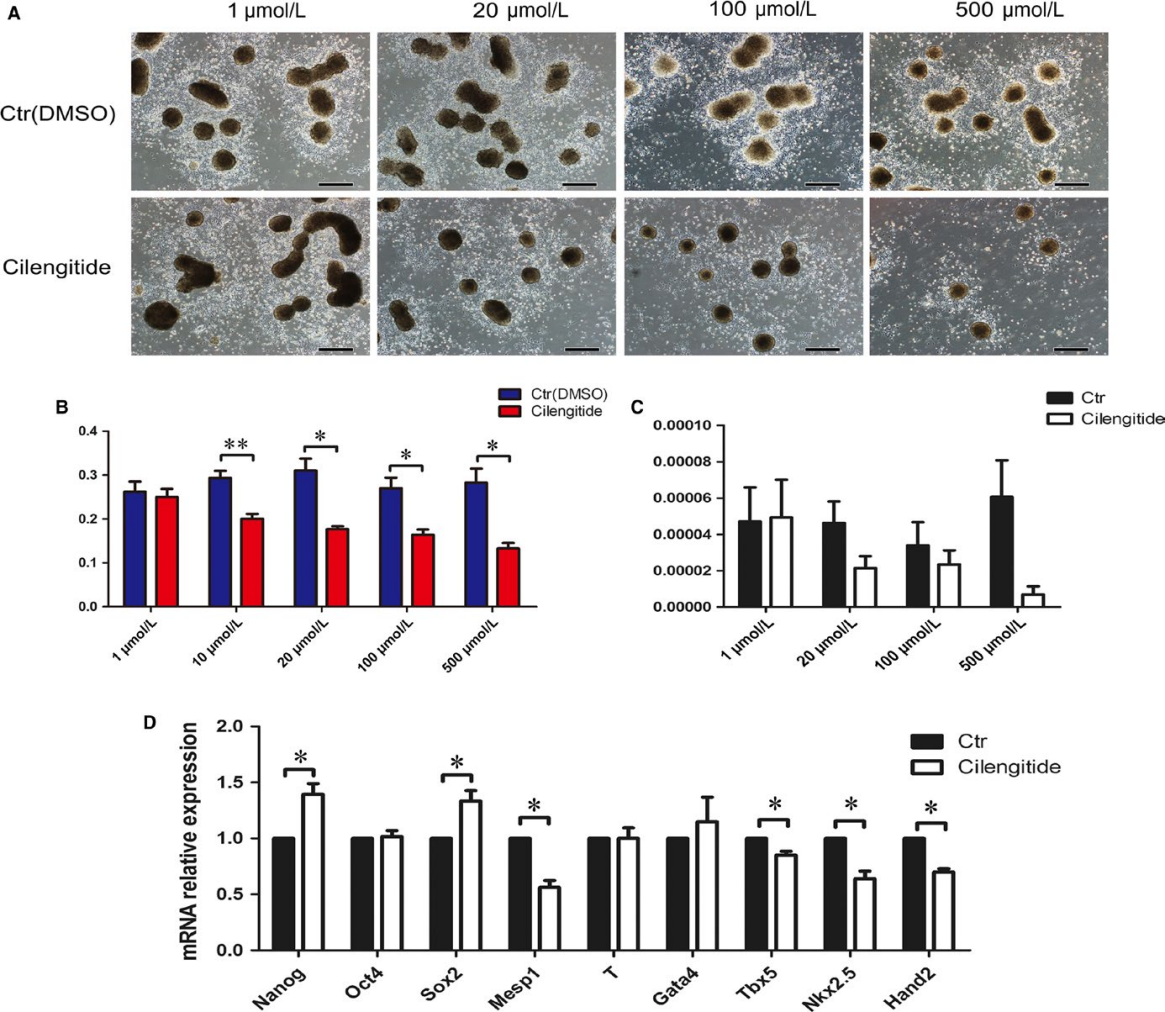
Effects of Cilengitide on rEBs proliferation and differentiation. A, The cellular morphology treated with Cilengitide at differentiation day 4 (scale bar: 500 μm). B, The cell proliferation findings determined by the CCK‐8 assay (n = 5), mean ± SEM, *P < 0.05, **P < 0.01. C, The Mesp1 mRNA expression levels at day 4 (n = 3), mean ± SEM. D, Expression of the differentiation‐related genes (n = 3), mean ± SEM, *P < 0.05

**Figure 5:**
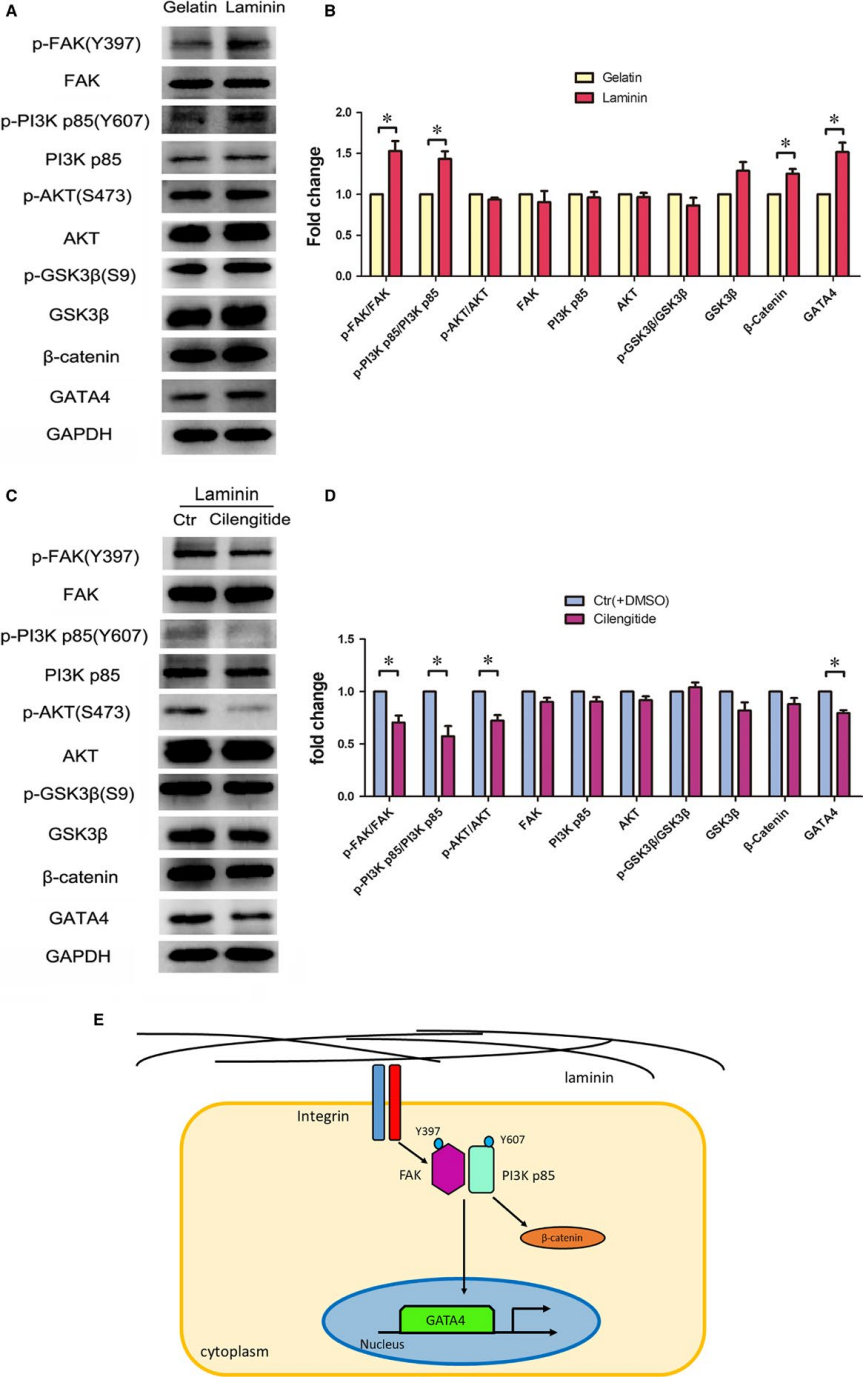
Activation of the integrin/PI3K/FAK pathway proteins. A and C, The protein, as well as the phosphorylated levels of the proteins, participating within the integrin/PI3K/FAK pathway. B and D, The quantification of (A) and (C) (n = 3), mean ± SEM, *P < 0.05. E, Proposed working model for the underlying regulatory cardiomyogenic mechanism in rESCs mediated by laminin

Integrin as a receptor of laminin plays a significant role in embryonic development.^28^ For example, integrin a6A interactions with laminin contribute to the development of myocardium at an early stage of heart development.^29^ To elucidate the details of how laminin facilitated differentiation of cardiomyocytes of rESCs, rEBs plated on laminin after droplet formation were treated with Cilengitide, a cyclic peptide which inhibits the integrin signalling through blocking integrin αvβ3 and αvβ5.^30^ The results showed that the number of rEB‐differentiated adherent cells gradually decreased with the increased dosage of Cilengitide (Figure 4A). In addition, the CCK‐8 proliferation assay at day 4 rEBs provided further evidence for the specific inhibition in a dose‐dependent manner (Figure 4B). As expected, the expression level of Mesp1 was also down‐regulated (Figure 4C). We next detected the expression status of differentiation‐related genes at day 4 of the differentiation in the presence of 30 μmol/L Cilengitide and found that Nanog and Sox2 were up‐regulated while the expression of the cardiac‐related genes Mesp1, Tbx5, Nkx2.5 and Hand2 were suppressed (Figure 4D). In addition, the laminin ‐mediated integrin interactions resulted in an elevation of the phosphorylation level of both FAK and PI3K p85 and in parallel β‐catenin and GATA4 were up‐regulated (Figure 5A,B). By inhibiting integrin with Cilengitide, laminin failed to stimulate the phosphorylation of p‐FAK, p‐PI3K p85, p‐AKT and to induce the expression of GATA4 (Figure 5C,D). These findings suggest that the cardiomyogenic effects of laminin are trigged via the Integrin/PI3K/FAK signalling pathway (Figure 5E).

### 3.5 The expression profiles of the genes related to cardiomyocyte differentiation in DIF/laminin

**Figure 6:**
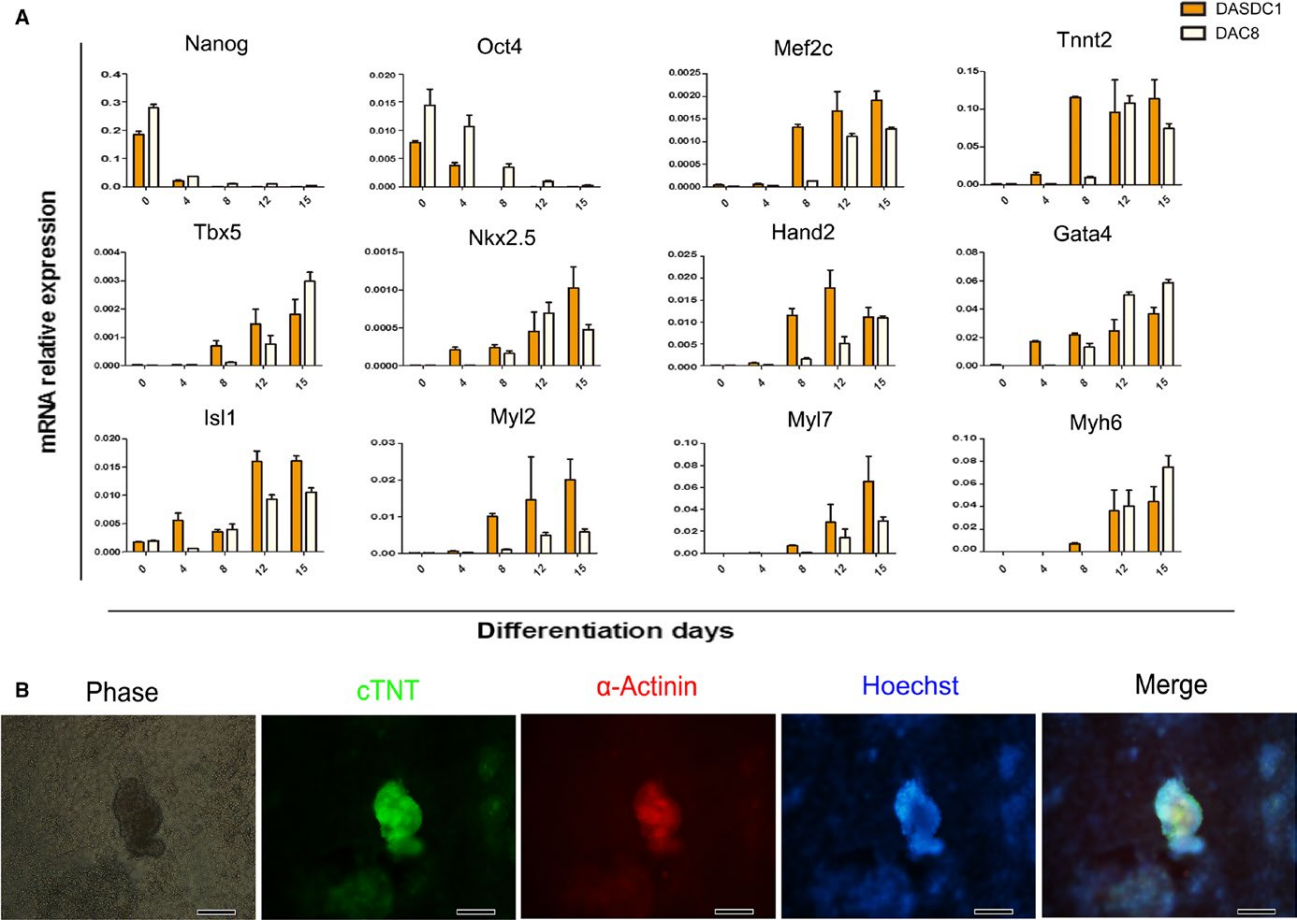
The expression profiles of genes related to cardiomyocyte differentiation. A, The mRNA expression levels during rEBs differentiation in different rESC lines (n = 3), mean ± SEM. B, Immunofluorescence results in 3D cell clusters of cTNT and α‐actinin at day 8 (scale bar: 100 μm)

To check the applicability of the differentiation conditions, the rESC lines DASDC1 and DAC8 were also used to induce differentiation under the DIF/Laminin conditions. As shown, in Figure 6A the expression of the pluripotent genes, Nanog and Oct4 were significantly inhibited from day 4 of differentiation and genes associated with cardiomyocyte differentiation, for example, Mef2c, Tbx5, Hand2, Nkx2.5, Gata4 and Isl1 were gradually up‐regulated from day 8 (Figure 6A). Moreover, the expression of mature functional cardiomyocyte genes such as Tnnt2, Myh6, Myl2 and Myl7, reached the highest expression level at day12‐day15 in different rESC lines (Figure 6A). Interestingly, some 3D‐like cell clusters which expressed cTNT and α‐Actinin markers began to appear from day 8 in this differentiation condition (Figure 6B and Figure S2). These results suggest that DIF in combination with laminin is optimal for the differentiation of rESC to cardiomyocytes. Meanwhile, the differentiation system was tested in rESC line DASDC2, which also confirmed the effect of cardiomyocyte differentiation by immunofluorescence assay (Figure S3).

## 4 DISCUSSION

It is well known that the differentiation of ESCs into cardiomyocyte lineage is co‐ordinated by a series of signalling pathways. In this context, the interplay between the WNT, Activin and BMP4 signalling is critical of cardiac‐lineage commitment in mESCs and hESCs to mesodermal cells.^31^, ^32^ FBS in certain concentration (eg 15%) is sufficient to stimulate mESC differentiation into cardiomyocytes without any external differentiating factors,^33^ indicating that FBS is essential for mediating cardiomyogenesis. However, there are some differences in the culture conditions between human embryonic stem cell (hESCs) differentiation and mESCs. The protocol for embryoid body‐based serum‐free culturing conditions have been confirmed as highly effective for orchestrating cardiomyocyte differentiation.^34^, ^35^, ^36^ Within this context, WNT, Activin and BMP4 were critical for the formation of hEBs and significantly improvement cardiomyogenesis in mESCs.^34^, ^37^, ^38^ However, fibroblast growth factor 2 (FGF2) plays a key role for the pluripotent and pre‐differentiation stages of hESC but not for the pluripotent stages in mESC.^39^ Interestingly, high concentration of FBS (20%) significantly improved the hESC differentiation potential when added after the mesoderm formation,^24^ suggesting that low concentrations of FBS are required at the early stage of differentiation of hESCs, while high levels of FBS are required at the mesoderm phase for an efficient improvement of cardiomyogenesis. In contrast to mESC and hESC,^6^ WNT, FGF and especially the MAPK signalling pathway are essential for maintaining of pluripotency of rESCs. These observations indicate that the in vitro differentiation conditions of both mESC and hESC are not fully applicable for rESC differentiation. For example, we found that high levels of FBS induced cell death of rESCs during rEBs aggregation, and rEBs failed to enter into the differentiation if the concentration of 2i was too high. All of these results suggest that the interactions of signal pathways by the addition of FBS with 2i need to reach a balance before rESC start to differentiate. We, hereby, optioned the necessary 2i concentration to maintain both stem cell activity and the survival ability. An increase of FBS concentration in the medium along with mesoderm formation can improve the cardiomyocyte differention. Ascorbic acid, a water‐soluble antioxidant widely used to improve the efficiency of cardiomyocyte differentiation, was also applied to enhance cardiomyogenesis of ESCs.^40^, ^41^ Overall, we developed the fundamental culture conditions, which efficiently promote rESCs differentiation into cardiomyocyte.

ECM as the major cellular microenvironment provides proteins and structures to support the stem cells to survive and further proliferates to undergo differentiation.^42^ To define the optimal culture conditions for cardiomyocyte differentiation in rESCs, we simulated the in vivo microenvironment by choosing the appropriate matrix. The basic components of the ECM include structural proteins (collagens), adhesion glycoproteins (laminin and fibronectin) and proteoglycans.^43^ Recently there is increasing evidence confirming the pivotal role of ECM for the development and function of the heart as it provides the scaffold for cardiomyocytes, endothelial cells, leukocytes, vascular smooth muscle cells and cardiac fibroblasts, resulting in the complex 3D heart structure. In this context, it is well‐established that ECM triggers intracellular signals to regulate cardiomyocytes and cardiac fibroblasts formation.^44^ For example, the ECM obtained by de‐cellularization of human heart tissue has been shown to enhance the differentiation efficiency of pluripotent stem cells to cardiac‐like cells with high expression levels of Nkx2.5, Myh6 and Tnnt2 thereby supporting cardiac lineage commitment.^45^ In addition, hyaluronan as one of the components of the ECM mediated the myofibroblast differentiation induced with transforming growth factor (TGF)‐β.^44^ Although matrigel and geltrex serving as cell scaffolds promote the differentiation of human pluripotent stem cells into cardiomocytes, matrigel only plays a role to maintain pluripotency of induced pluripotent stem cells from mouse.^45^ Why matrigel and geltrex exert distinct effects on cardiomyogenesis of hESC and mESC? The matrigel obtained from Engelbreth‐Holm‐Swarm (EHS) mouse sarcoma is composed of laminin (60%), collagen IV (30%), heparan sulphate proteoglycans (8%) and entactin or nidogen (1%).^46^ Also the commercial geltrex matrix contains laminin, collagen IV, entactin and heparin sulphate proteoglycans. All of these distinct components of the ECM may contribute to trigger specific signalling pathways involved in cardiomyocyte differentiation of hESCs and mESCs. Moreover, gelatin, a mixture of peptides and proteins extracted from the skin, bones and connective tissues of animals has been used as ECM to induce the differentiation of mESCs or hESCs into cardiomyocytes.^20^, ^47^ Indeed, we provided evidence that laminin is the critical component of ECMs required for an optimal and efficient differentiation of rESC to cardiomyocytes.

The signalling pathways involved in the rESC differentiation to cardiomyocytes are still elusive. The key signalling factors that maintain pluripotent status in rESC have been found to be different compared with that in hESC and mESC.^48^ Unlike the adherent growth of both mESCs and hESCs, the rESC growth in suspension suggest that the cells may have distinct surface receptors in comparison with that of hESCs and mESCs. As shown in this study, we found that laminin is conducive in promoting rESCs differentiation into cardiomyocytes by activating integrin and its downstream PI3K p85 and FAK signalling pathways. In this context, we demonstrated AKT was phosphorylated at S473 by FAK Y397 and PI3K p85 Y607, and GATA4 was up‐regulated to induce cardiomyogenesis indicating the involvement of integrin as the receptor of the laminin‐induced cardiomyocyte cardiomyogenesis in rESCs. In contrast to rESCs, phosphorylation of FAK and AKT via integrin activation is required to maintain the hESC pluripotency.^49^, ^50^ On the other hand, the interplay of integrin α6β1 with laminin is required for mESC differentiation into endothelial cells.^51^ The unique features of the signalling pathways for pluripotency and differentiation stage differ significantly between the hESCs, mESCs and rESCs.

Organoids with 3D structures of different mature cell types can be derived from ESCs under appropriate conditions,^52^ and show organ‐like structural features, such as cell polarity, junctions and interactions with ECM.^53^ Reliable methods are increasing for generating organoids for intestine, brain, lung, eyes, stomach and kidney.^54^ Here, we generated 3D clusters of cardiomyocytes in the differentiation system with PD03 and CHIR and verified the roles of WNT, MEK and integrin/FAK/PI3K/AKT pathway in the process, providing a potential condition for generating rat cardiac organoids.

## 5 CONCLUSIONS

We established the high efficiency system for cardiomyocyte differentiation or even formation of 3D‐like cell clusters expressed with cTNT and α‐Actinin from rESCs, and further eluciated that laminin significantly improve rEB attachment and the proliferation of the differentiating cells. Moreover, the integrin/FAK/PI3K pathway was found to be activated to mediate the rESC‐derived cardiomyocyte or 3D‐like clusters. Our results provide an in vitro platform to understand the details of early stage in heart development.

## CONFLICT OF INTEREST

The authors indicated no potential conflicts of interest.

## AUTHORS’ CONTRIBUTIONS

L. Peng and D. Wang conceived the research; L. Peng and D. Wang designed the experiment; D. Wang performed the experiments and analysed the data; all authors discussed the manuscript; D. Wang wrote the manuscript; L. Peng, Q. Ying, A. Sachinidis, Y. Wang and L. Li revised the manuscript; L. Peng and L. Li supervised the project.
